# The 3rd London General Hospital

**Published:** 1915-11-06

**Authors:** H. Rowland Brown

**Affiliations:** Secretary for School. 17 Waterloo Place, London, S.W.


### THE EDITOR'S LETTER-BOX.

### The 3rd London General Hospital.

THE ROYAL PATRIOTIC FUND CORPORATION AND THE ROYAL VICTORIA PATRIOTIC SCHOOL.

*To the Editor of* THE HOSPITAL.

SIR,—My attention has been called to the interesting article in THE HOSPITAL for October 23, dealing with the 3rd London General Hospital, Wandsworth Common, London, S.W.

I wish to point out to you that the first paragraph of this article is incorrect. The buildings now in occupation of No. 3 General Hospital are not the permanent buildings of the Royal Patriotic Fund Corporation. These buildings are those of the Royal Victoria Patriotic School, which belongs to the Royal Patriotic Fund Corporation. The offices of this Corporation are at 17 Waterloo Place, S.W. The buildings were taken over by the War Office under the Defence of the Realm Act on August 4, 1914; the children thus displaced have been installed in the neighbourhood of Wandsworth Common in a number of houses on the Spencer Park Estate and North Side.

I would ask you kindly to make a note of these several statements in your next issue as the paragraph, as it stands, is somewhat misleading.—Yours very faithfully,

H. ROWLAND BROWN, Secretary for School.

17 Waterloo Place, London, S.W.
October 28, 1915.

[At the request of our correspondent we publish his letter. We have not been able to appreciate its point; perhaps our readers may be more fortunate.—ED. THE HOSPITAL.]

### "Frost Bite" in the Trenches.

*To the Editor of* THE HOSPITAL.

